# Metabolically defined body size and body shape phenotypes and risk of postmenopausal breast cancer in the European Prospective Investigation into Cancer and Nutrition

**DOI:** 10.1002/cam4.5896

**Published:** 2023-04-25

**Authors:** Y. Mahamat‐Saleh, S. Rinaldi, R. Kaaks, C. Biessy, E. M. Gonzalez‐Gil, N. Murphy, C. Le Cornet, J. M. Huerta, S. Sieri, A. Tjønneland, L. Mellemkjær, M. Guevara, K. Overvad, A. Perez‐Cornago, S. Tin Tin, L. Padroni, V. Simeon, G. Masala, A. May, E. Monninkhof, S. Christakoudi, A. K. Heath, K. Tsilidis, A. Agudo, M. B. Schulze, J. Rothwell, C. Cadeau, S. Severi, E. Weiderpass, M. J. Gunter, L. Dossus

**Affiliations:** ^1^ International Agency for Research on Cancer Lyon France; ^2^ Division of Cancer Epidemiology German Cancer Research Center (DFKZ) Heidelberg Germany; ^3^ Department of Epidemiology Murcia Regional Health Council Murcia Spain; ^4^ CIBER Epidemiología y Salud Pública (CIBERESP) Madrid Spain; ^5^ Epidemiology and Prevention Unit Fondazione IRCCS Istituto Nazionale dei Tumori 20133 Milan Italy; ^6^ Danish Cancer Society Research Center Copenhagen Denmark; ^7^ Department of Public Health University of Copenhagen Copenhagen Denmark; ^8^ Navarra Public Health Institute 31003 Pamplona Spain; ^9^ Consortium for Biomedical Research in Epidemiology and Public Health (CIBERESP) 28029 Madrid Spain; ^10^ Navarra Institute for Health Research (IdiSNA) 31008 Pamplona Spain; ^11^ Department of Public Health, Section for Epidemiology Aarhus University Aarhus Denmark; ^12^ Cancer Epidemiology Unit Nuffield Department of Population Health University of Oxford Oxford UK; ^13^ Department of Clinical and Biological Sciences University of Turin Turin Italy; ^14^ Dipartimento di Salute Mentale e Fisica e Medicina Preventiva Università degli Studi della Campania 'Luigi Vanvitelli' 80121 Naples Italy; ^15^ Institute for Cancer Research, Prevention and Clinical Network (ISPRO) Florence Italy; ^16^ Julius Center for Health Sciences and Primary Care University Medical Center Utrecht Utrecht University Utrecht The Netherlands; ^17^ Department of Epidemiology and Biostatistics School of Public Health, Imperial College London London UK; ^18^ Department of Inflammation Biology School of Immunology and Microbial Sciences King's College London London UK; ^19^ Unit of Nutrition and Cancer Catalan Institute of Oncology – ICO L'Hospitalet de Llobregat Spain; ^20^ Nutrition and Cancer Group; Epidemiology, Public Health, Cancer Prevention and Palliative Care Program Bellvitge Biomedical Research Institute – IDIBELL L'Hospitalet de Llobregat Spain; ^21^ Department of Molecular Epidemiology German Institute of Human Nutrition Potsdam‐Rehbruecke Nuthetal Germany; ^22^ Institute of Nutritional Science University of Potsdam Nuthetal Germany; ^23^ Paris‐Saclay University UVSQ, Inserm, Gustave Roussy, “Exposome and Heredity” team, CESP Villejuif France

**Keywords:** body mass index, breast cancer, concentrations of C‐peptide, metabolic health, waist circumference, waist‐to‐hip ratio

## Abstract

**Background:**

Excess body fatness and hyperinsulinemia are both associated with an increased risk of postmenopausal breast cancer. However, whether women with high body fatness but normal insulin levels or those with normal body fatness and high levels of insulin are at elevated risk of breast cancer is not known. We investigated the associations of metabolically defined body size and shape phenotypes with the risk of postmenopausal breast cancer in a nested case–control study within the European Prospective Investigation into Cancer and Nutrition.

**Methods:**

Concentrations of C‐peptide—a marker for insulin secretion—were measured at inclusion prior to cancer diagnosis in serum from 610 incident postmenopausal breast cancer cases and 1130 matched controls. C‐peptide concentrations among the control participants were used to define metabolically healthy (MH; in first tertile) and metabolically unhealthy (MU; >1st tertile) status. We created four metabolic health/body size phenotype categories by combining the metabolic health definitions with normal weight (NW; BMI < 25 kg/m^2^, or WC < 80 cm, or WHR < 0.8) and overweight or obese (OW/OB; BMI ≥ 25 kg/m^2^, or WC ≥ 80 cm, or WHR ≥ 0.8) status for each of the three anthropometric measures separately: (1) MHNW, (2) MHOW/OB, (3) MUNW, and (4) MUOW/OB. Conditional logistic regression was used to compute odds ratios (ORs) and 95% confidence intervals (CIs).

**Results:**

Women classified as MUOW/OB were at higher risk of postmenopausal breast cancer compared to MHNW women considering BMI (OR = 1.58, 95% CI = 1.14–2.19) and WC (OR = 1.51, 95% CI = 1.09–2.08) cut points and there was also a suggestive increased risk for the WHR (OR = 1.29, 95% CI = 0.94–1.77) definition. Conversely, women with the MHOW/OB and MUNW were not at statistically significant elevated risk of postmenopausal breast cancer risk compared to MHNW women.

**Conclusion:**

These findings suggest that being overweight or obese and metabolically unhealthy raises risk of postmenopausal breast cancer while overweight or obese women with normal insulin levels are not at higher risk. Additional research should consider the combined utility of anthropometric measures with metabolic parameters in predicting breast cancer risk.

## Graphical abstract

In this nested case‐control study, we found that being overweight or obese and metabolically unhealthy raises risk of postmenopausal breast cancer while overweight or obese women with normal insulin levels are not at higher risk. These findings are consistent with findings from previous cohort studies.

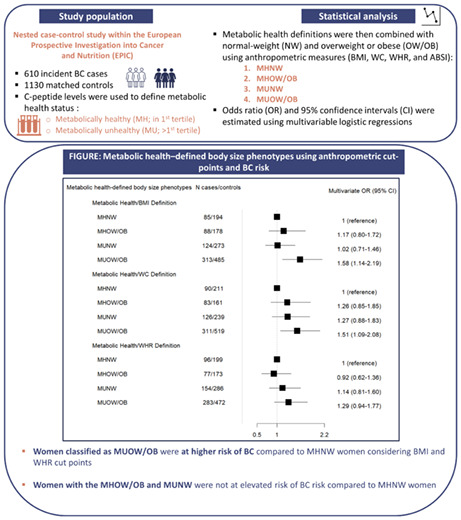

## 1 INTRODUCTION

Excess body fatness is a major risk factor for several chronic diseases, including type 2 diabetes,^1^ cardiovascular diseases,^2^ and certain types of cancer.^3^, ^4^ Overweight and obesity, assessed through various anthropometric measures, are well‐established risk factors for postmenopausal breast cancer^3^, ^4^—the most common cancer diagnosed in women, the incidence of which has increased considerably over the past decades.^5^ Adiposity may trigger the development of postmenopausal breast cancer through increased levels of sex steroid hormones, insulin resistance, oxidative stress, and inflammation.^4^ Numerous prospective cohort studies have indeed reported that elevated levels of fasting insulin or C‐peptide, a marker for insulin secretion, were associated with higher breast cancer incidence^6^, ^7^, ^8^, ^9^, ^10^ and experimental studies have shown that insulin may lead to breast cancer development through its mitogenic and antiapoptotic activity.^11^, ^12^ A recent Mendelian randomization study reported that genetically predicted fasting insulin and glucose levels were positively associated with breast cancer risk.^13^ In addition, increasing evidence suggests that diabetes and the metabolic syndrome (MetS) may increase the risk of breast cancer.^14^, ^15^ Recently, studies using a mediation analysis approach showed that fasting insulin plays a major role in explaining the adiposity–postmenopausal breast cancer association—attributing 58%–65.8% of the association to insulin pathways.^6^, ^16^, ^17^ However, despite adiposity and insulin resistance having both been linked to an increased risk of postmenopausal breast cancer, whether women with high body fatness who have normal insulin sensitivity [a low level of homeostasis model assessment of insulin resistance (HOMA‐IR) or circulating insulin or C‐peptide levels], or those with normal body fatness who have elevated levels of insulin are at risk of breast cancer is not well‐documented.

Metabolic obesity phenotypes have been recently investigated in relation to obesity‐related outcomes.^18^, ^19^, ^20^, ^21^, ^22^, ^23^, ^24^, ^25^, ^26^, ^27^, ^28^, ^29^ There is now accumulating evidence suggesting that individuals who are overweight or obese and have insulin resistance, defined as metabolically unhealthy, are at elevated risk of cardiovascular disease, compared to normal‐weight individuals with normal insulin levels; however, those who are overweight or obese, but who have normal insulin sensitivity (defined as metabolically healthy) have little risk.^21^, ^24^, ^25^, ^26^ With regards to incident cancer risk, previous studies suggested that metabolically unhealthy participants who are normal weight or overweight had higher risks of cancers of the colorectum,^30^ prostate,^31^ bladder,^32^ pancreas,^33^ and endometrium^34^ compared to those who are metabolically healthy with normal weight.

To date, only four prospective US studies have investigated the role of metabolic obesity phenotypes in relation to postmenopausal breast cancer risk.^19^, ^35^, ^36^, ^37^ These studies suggested that being overweight or obese and metabolically unhealthy was associated with an increased risk of postmenopausal breast cancer compared to metabolically healthy normal weight women, whereas women with metabolically healthy overweight or those with metabolically unhealthy normal weight were not at elevated risk of breast cancer.^19^, ^35^, ^36^, ^37^ However, three of these past studies did not examine associations according to potential effect modifiers such as use of oral contraceptive or menopausal hormone therapy, circulating estradiol levels and age at diagnosis. In addition, only two studies assessed the association of metabolic dysfunction with central adiposity using waist circumference (WC) and waist‐to‐hip ratio (WHR) definitions.

In this analysis, we sought to investigate the associations of metabolically defined body size and shape phenotypes with the risk of postmenopausal breast cancer in a case–control study nested within the European Prospective Investigation into Cancer and Nutrition (EPIC).

## 2 MATERIALS AND METHODS

### 2.1 EPIC participants

EPIC is an ongoing multi‐centric prospective cohort study designed to assess the relationship between diet, lifestyle, genetic and metabolic factors and the incidence of cancer and other chronic diseases.^38^ Briefly, the cohort involves over 153,000 men and 368,000 women aged 35–75 years at inclusion and recruited between 1992 and 2000 in 23 centers in 10 European countries (France, Denmark, Germany, Greece, Italy, the Netherlands, Norway, Spain, Sweden, and the United Kingdom). The rationale, study design and methods have been described in detail elsewhere.^38^, ^39^ The study was approved by the local ethical committees in participating countries and the International Agency for Research on Cancer (IARC) ethical committee. All participants provided written informed consent before study entry.

### 2.2 Follow‐up and identification of incident breast cancer cases

The identification of incident cancers was conducted using record linkage with population‐based cancer registries in Denmark, Italy, the Netherlands, Spain, Sweden, and the United Kingdom. In contrast, in France and Germany, cancer cases during follow‐up were identified through a combination of several methods including health insurance records, contacts with cancer and pathology registries, and active follow‐up of participants and their next of kin. During follow‐up, data on vital status in most EPIC study centers were obtained from cancer or mortality registries at the regional or national level. Information on molecular status and stage of breast cancer was collected from each center, where possible.

### 2.3 Selection of case and control subjects

This present study uses data from a previous nested case–control study in which serum C‐peptide levels were measured in 1141 incident breast cancer cases and 2204 matched controls from seven EPIC countries.^10^ Participants from Greece were excluded due to unavailability of data, and those from Norway were initially excluded because biological samples were not completely collected and there were very few cases of breast cancer diagnosed after blood collection when the laboratory analyses were undertaken. Participants from Sweden were not selected because the association between insulin levels and breast cancer had been assessed separately within that population when the project started.^40^

Case participants were identified among women diagnosed with breast cancer after the initial blood collection and before the end of the follow‐up in each study center. At the time of blood collection, women who had a prevalent cancer (excluding keratinocyte cancer) and those who used any menopausal hormone therapy or any exogenous hormones for contraception or medical purposes were excluded from this study; however, those who used exogenous hormones in the past were not excluded from this analysis. Women who did not provide a blood sample or those with missing information on fasting status and serum C‐peptide were also excluded.

For each breast cancer case, two controls were randomly selected among women who were alive and free of cancer (except keratinocyte cancer) at the time of diagnosis of the index case. An incidence density sampling protocol for control selection was used, such that controls could include participants who became a case later in time, while each control could also be sampled more than once. Controls were matched to cases on study center, age at blood collection (continuous), menopausal status (premenopausal, perimenopausal, postmenopausal, surgically postmenopausal), time of day at blood collection (± 1 h), and fasting status at blood collection (non‐fasting (<3 h since last meal), in between (3–6 h), fasting (>6 h), unknown).

In addition to the previous exclusions, we excluded pre and perimenopausal cases and their matched controls leaving a final study sample of 610 incident postmenopausal breast cancer cases and 1130 matched controls with available information for analysis.

### 2.4 Laboratory measurements

Blood samples were collected at baseline according to standardized procedures and stored at the IARC (−196°C, liquid nitrogen) for all countries except Denmark (−150°C, nitrogen vapor), and Sweden (at − 80°C in standard freezers).^38^, ^39^ Concentrations of C‐peptide were measured in serum samples in the laboratories of the Nutrition and Metabolism Branch at IARC by radioimmunoassay from Diagnostic Systems Laboratories (DSL, Webster, Texas).^10^ As previously reported, samples from matched case–control sets were analyzed in the same analytical batch, and all laboratory personnel performing the assay were blinded to the case–control status of the samples. The mean intra‐batch and inter‐batch coefficients of variation were 6.7% and 9.8%, respectively.^10^ In addition, estradiol and estrone concentrations in serum were also measured by radioimmunoassays at IARC.^41^

### 2.5 Assessment of anthropometric, lifestyle, and dietary exposures

Data on lifestyle and dietary intake were collected at recruitment through questionnaires in all centers.^38^ Body weight (kg) and height (cm) were measured using standardized procedures without shoes in all centers except for EPIC Oxford and France where anthropometric measures were self‐reported. Body mass index (BMI) was calculated by dividing weight (kg) by square of the height (m). Waist circumference (WC; cm) was measured at the narrowest torso circumference or at the midpoint between the lower ribs and iliac crest depending on study center. Hip circumference (cm) was measured at the level of the largest lateral extension of the hips or over the buttocks. To calculate the waist‐to‐hip ratio (WHR), waist circumference was divided by hip circumference.

Dietary intake including alcohol intake over the 12 months before recruitment were assessed using validated country‐specific dietary and lifestyle questionnaires designed to reflect local dietary habits.^42^ Detailed information on other lifestyle and medical history factors such as education, smoking status, physical activity, diabetes, and reproductive factors (menopausal status, menopausal hormone use, age at menarche and menopause, and age and number of full‐term pregnancies) was obtained using gender‐specific questionnaires in all centers.^39^, ^43^

### 2.6 Definition of metabolic health and body size categories

To define metabolic health, we used concentrations of C‐peptide among the control participants as previously done in EPIC studies on other cancer sites.^30^, ^34^ We classified women as metabolically healthy if they were in the first tertile of C‐peptide concentration (<2.76 ng/mL) and metabolically unhealthy if they had concentration of C‐peptide above the first tertile (≤2.76 ng/mL). To compare findings with the previous studies, the same procedure was performed using quartiles (first quartile, 2.02 ng/mL as metabolically healthy) and median values (below median, 3.42 ng/mL as metabolically healthy) of C‐peptide concentration among the control participants.

To generate metabolic health/body size and shape phenotype categories, women were classified into four groups by cross‐tabulating anthropometric measures (one at a time) with metabolic health status: (1) metabolically healthy and normal weight (BMI < 25 kg/m^2^, or WC < 80 cm, or WHR < 0.80; MHNW); (2) metabolically healthy and overweight/obese (BMI ≥ 25 kg/m^2^, or WC ≥ 80 cm, or WHR ≥ 0.80; MHOW/OB); (3) metabolically unhealthy and normal weight (BMI < 25 kg/m^2^, or WC < 80 cm, or WHR < 0.80; MUNW); (4) metabolically unhealthy and overweight/obese (BMI≥25 kg/m^2^, or WC ≥ 80 cm, or WHR≥0.80; MUOW/OB). We considered the metabolically healthy/normal weight (MHNW) status as the reference group.

### 2.7 Statistical analysis

Statistical analyses were performed using the SAS package (version 9.4, SAS Institute). We first compared the baseline characteristics between cases and controls using mean and standard deviation (SD), for continuous data, or frequency distributions for categorical variables. We then performed descriptive analyses according to metabolically defined body size and shape phenotypes among the controls.

Odds ratios (ORs) and 95% confidence intervals (CIs) for the associations between metabolically defined body size and shape phenotypes and breast cancer risk were estimated using conditional logistic regression. Model 1 included matching criteria only. The multivariable models were further adjusted for known breast cancer risk factors including age at menarche (> 13 and ≤13 years), number of full‐term pregnancies and age at first full‐term pregnancy (nulliparous; age < 30 years (1–2 children); age < 30 years (≥3 children) and age years ≥ 30), age at menopause (continuous), breastfeeding (no; yes), previous use of oral contraceptive pills (no; yes), previous use of menopausal hormonal therapy (MHT; no; yes), physical activity index (inactive; moderately inactive; moderately active; active), alcohol consumption (non‐drinker; 0.1 to ≤3; 3.1 to ≤12; 12.1 to ≤24 and >24), smoking status (never; former smoker; current smoker), educational level (primary/no schooling; technical/professional/secondary and longer education; missing), height (continuous), and energy intake from diet (continuous; Model 2). For all adjustment variables, if values were missing in <5% observations, they were imputed to the median, for continuous variables, or mode, for categorical variables and a “missing” category (for education level only) was introduced otherwise.

We evaluated heterogeneity of the associations by fasting status, previous use of oral contraceptives, circulating levels of estradiol or estrone and age at breast cancer diagnosis (<60 and ≥60 years). Heterogeneity tests were assessed using Wald chi‐square tests to compare estimates across strata. In addition, we performed sensitivity analyses by (1) excluding women who reported a history of diabetes, which may affect C‐peptide concentration, (2) restricting analyses to women who had never used menopausal hormone therapy (3) considering only the upper tertile of C‐peptide as metabolically unhealthy group, (4) exploring the phenotypes defined based on quartiles or on median level of C‐peptide cut points and (5) using a cutoff of 88 cm and 0.88 for WC and WHR, respectively. In addition, we assessed separately the association between each of the components of metabolically defined body size phenotypes and the risk of postmenopausal breast cancer. To explore a potential reverse‐causation bias, we assessed the association between metabolically defined body size phenotypes and breast cancer risk by excluding cases (*N* = 209) diagnosed within the first 2 years of follow‐up and their matched controls (*N* = 384). Since information on breast cancer subtypes was missing for a large proportion of breast cancer cases, we were not able to explore breast cancer risk according to molecular subtypes.

## 3 RESULTS

### 3.1 Characteristics of study participants

Overall, 610 incident postmenopausal breast cancer cases and 1130 matched controls were included in this study. Briefly, cases were diagnosed on average at age 64 years and the average time from blood collection to breast cancer diagnosis was 3 years. Compared to controls, breast cancer cases had slightly higher WC and serum levels of C‐peptide, and were more likely to be non‐alcoholic drinkers, to report a history of hypertension and to have higher total energy intakes. However, they were less likely to be current smokers and to report previous use of oral contraceptive or MHT. Cases and controls were similar with respect to height, BMI, education, physical activity, diabetes, and other reproductive and menstrual variables (Table 1).

**TABLE 1:** Baseline characteristics of study participants according to breast cancer status.

Baseline characteristics	Case (N = 610)	Control (N = 1130)
	Mean (SD) or *N* (%)	Mean (SD) or *N* (%)
Time between blood collection and diagnosis in years	3.02 (2.0)	—
Age at diagnosis in years	63.55 (5.8)	—
Age at blood collection in years	60.53 (5.5)	60.44 (5.4)
Anthropometric factors		
Height in cm	160.71 (6.4)	160.00 (6.7)
Body mass index in kg/m^2^	26.86 (4.3)	26.56 (4.6)
Waist circumference in cm	84.42 (10.8)	83.47 (10.8)
Waist‐to‐hip ratio	0.81 (0.1)	0.81 (0.1)
Sociodemographic, lifestyle, and reproductive factors		
Education level		
Primary/no schooling	305 (50.0)	562 (49.7)
Technical/professional/secondary	209 (34.3)	402 (35.6)
Longer education	60 (9.8)	106 (9.4)
Missing	36 (5.9)	60 (5.3)
Fasting status at blood collection		
Non‐fasting	338 (55.4)	626 (55.4)
In between	80 (13.1)	146 (12.9)
Fasting	192 (31.5)	358 (31.7)
Physical activity index		
Inactive	176 (28.8)	333 (29.5)
Moderately inactive	219 (35.9)	395 (34.9)
Moderately active	106 (17.4)	198 (17.5)
Active	109 (17.9)	204 (18.1)
Smoking status		
Never	386 (63.3)	674 (59.7)
Former	141 (23.1)	283 (25.0)
Current	83 (13.6)	173 (15.3)
Alcohol consumption at recruitment in g/day		
Non drinker	146 (23.9)	256 (22.7)
0.1 to ≤3	187 (30.7)	384 (33.9)
3.1 to ≤12	144 (23.6)	272 (24.1)
12.1 to ≤24	79 (12.8)	137 (12.1)
>24	54 (9.0)	81 (7.2)
Age at menopause in years	49.47 (4.6)	49.02 (4.7)
Age at first menstrual period in years		
≤13	216 (35.4)	404 (35.7)
>13	394 (64.6)	726 (64.3)
Ever breastfed	447 (73.3)	825 (73.0)
Previous use of oral contraceptives	196 (32.1)	415 (36.7)
Previous use of menopausal hormone therapy	102 (16.7)	206 (18.2)
Number of full‐term pregnancies and age at first full‐term pregnancy		
Nulliparous	79 (13.0)	163 (14.4)
Age < 30 (1–2 children)	265 (43.4)	424 (37.5)
Age < 30 (≥3 children)	171 (28.0)	386 (34.2)
Age ≥ 30	95 (15.6)	157 (13.9)
Comorbidities		
History of diabetes		
No	488 (80.0)	903 (79.9)
Yes	20 (3.3)	41 (3.6)
Missing	102 (16.7)	186 (16.5)
History of hypertension		
No	215 (35.3)	413 (36.5)
Yes	176 (28.8)	285 (25.2)
Missing	219 (35.9)	432 (38.2)
Total energy intake in kcal/day	1919.8 (537.0)	1885.0 (553.8)
C‐peptide in ng/mL	4.34 (2.7)	4.07 (2.3)

*Note*: Controls were matched to cases on study center, age at blood collection, time of day at blood collection, and fasting status at blood collection.

Compared to MHNW women and considering the BMI definition, women classified as MUNW were younger at their first menstrual period and at blood collection, were taller, and had larger WC, and were more likely to have higher physical activity levels, and to report previous use of oral contraceptives and MHT. They were less likely to have higher education, to be current smokers and to consume alcohol. In contrast, MUOW/OB women were slightly younger at first menstrual period and at menopause and tended to have higher BMI, WC and WHR compared to MHNW women; but they had lower levels of education, physical activity, and total energy intake, and were less likely to be current smokers, to consume alcohol and to report previous use of oral contraceptives (Table 2). Results were globally similar for metabolically defined body size phenotypes based on WC or WHR definitions compared to those based on BMI definition (Table 2). C‐peptide levels were higher among MUOW/OB women compared to MHNW women. Globally, levels of C‐peptide in the four subgroups did not differ according to the anthropometric index used to define body size and body shape.

**TABLE 2:** Baseline characteristics of control participants according to metabolic health–defined body size phenotypes using anthropometric cut points in postmenopausal women.

	Total	Metabolic health/BMI definition (*N* = 1130)				Metabolic health/WC definition (*N* = 1130)				Metabolic health/WHR definition (*N* = 1130)			
		Metabolically healthy		Metabolically unhealthy		Metabolically healthy		Metabolically unhealthy		Metabolically healthy		Metabolically unhealthy	
		NW^a^	OW/OB^b^	NW^c^	OW/OB^d^	NW^a^	OW/OB^b^	NW^c^	OW/OB^d^	NW^a^	OW/OB^b^	NW^c^	OW/OB^d^
C‐peptide in ng/mL^*^	4.07 (2.3)	2.11 (0.5)	2.22 (0.4)	4.75 (2.1)	5.23 (2.6)	2.09 (0.5)	2.25 (0.4)	4.71 (2.2)	5.23 (2.5)	2.07 (0.4)	2.28 (0.4)	4.89 (2.4)	5.18 (2.5)
Age at blood collection in years	60.44 (5.4)	59.49 (5.5)	59.83 (4.8)	60.63 (5.5)	60.92 (5.5)	59.81 (5.5)	59.46 (4.8)	60.49 (5.7)	60.97 (5.4)	59.62 (5.3)	59.70 (5.1)	60.23 (5.1)	61.17 (5.7)
Anthropometric factors													
Height in cm	159.99 (6.7)	160.35 (6.0)	158.12 (6.5)	162.43 (6.6)	159.17 (6.7)	159.25 (5.8)	159.33 (6.9)	160.92 (6.3)	160.08 (7.1)	159.34 (6.0)	159.22 (6.7)	161.63 (6.2)	159.56 (7.1)
Body mass index in kg/m^2^	26.56 (4.6)	22.27 (1.7)	28.10 (2.6)	22.91 (1.6)	29.76 (4.2)	22.95 (2.4)	27.82 (3.1)	23.25 (2.3)	29.15 (4.5)	23.68 (3.0)	26.65 (3.7)	25.18 (3.7)	28.57 (4.9)
Waist circumference in cm	83.47 (10.8)	73.42 (5.7)	86.38 (8.4)	76.30 (5.9)	90.46 (9.7)	72.98 (4.6)	88.32 (7.2)	74.04 (4.0)	90.57 (8.9)	73.76 (5.8)	86.36 (8.6)	77.44 (6.9)	90.15 (10.0)
Waist‐to‐hip ratio	0.81 (0.07)	0.78 (0.07)	0.82 (0.06)	0.78 (0.06)	0.83 (0.06)	0.76 (0.06)	0.84 (0.05)	0.76 (0.04)	0.84 (0.06)	0.75 (0.03)	0.85 (0.06)	0.75 (0.03)	0.85 (0.05)
Sociodemographic, reproductive, and lifestyle factors													
Education level													
Primary/no schooling	562 (49.7)	72 (37.1)	118 (66.3)	92 (33.7)	280 (57.7)	85 (40.3)	105 (65.2)	84 (35.2)	288 (55.5)	562 (49.7)	83 (41.7)	107 (61.8)	109 (38.1)
Technical/professional/secondary	402 (35.5)	79 (40.7)	40 (22.5)	133 (48.7)	150 (30.9)	79 (37.4)	40 (24.8)	110 (46.0)	173 (33.3)	402 (35.5)	72 (36.2)	47 (27.2)	128 (44.8)
Longer education	106 (9.4)	32 (16.5)	8 (4.5)	34 (12.4)	32 (6.6)	32 (15.2)	8 (4.9)	31 (13.0)	35 (6.7)	106 (9.4)	29 (14.6)	11 (6.4)	32 (11.2)
Missing	60 (5.4)	11 (5.7)	12 (6.7)	14 (5.1)	23 (4.7)	15 (7.1)	8 (4.9)	14 (5.8)	23 (4.4)	60 (5.4)	15 (7.5)	8 (4.6)	17 (5.9)
Fasting status at blood collection													
Non‐fasting	626 (55.4)	79 (40.7)	58 (32.6)	211 (77.3)	278 (57.3)	79 (37.4)	58 (36.0)	180 (75.3)	309 (59.5)	626 (55.4)	82 (41.2)	55 (31.8)	215 (75.2)
In between	146 (12.9)	22 (11.4)	24 (13.5)	33 (12.1)	67 (13.8)	27 (12.8)	19 (11.8)	28 (11.7)	72 (13.9)	146 (12.9)	25 (12.6)	21 (12.1)	38 (13.3)
Fasting	358 (31.7)	93 (47.9)	96 (53.9)	29 (10.6)	140 (28.9)	105 (49.8)	84 (52.2)	31 (13.0)	138 (26.6)	358 (31.7)	92 (46.2)	97 (56.1)	33 (11.5)
Physical activity index													
Inactive	333 (29.5)	53 (27.3)	58 (32.6)	59 (21.6)	163 (33.6)	58 (27.5)	53 (32.9)	62 (25.9)	160 (30.8)	333 (29.5)	53 (26.6)	58 (33.5)	61 (21.3)
Moderately inactive	395 (34.9)	59 (30.4)	70 (39.3)	95 (34.8)	171 (35.3)	72 (34.1)	57 (35.5)	77 (32.3)	189 (36.4)	395 (34.9)	63 (31.7)	66 (38.2)	108 (37.8)
Moderately active	198 (17.5)	46 (23.7)	28 (15.7)	54 (19.8)	70 (14.4)	44 (20.9)	30 (18.6)	44 (18.4)	80 (15.4)	198 (17.5)	48 (24.1)	26 (15.0)	52 (18.2)
Active	204 (18.1)	36 (18.6)	22 (12.4)	65 (23.8)	81 (16.7)	37 (17.5)	21 (13.0)	56 (23.4)	90 (17.4)	204 (18.1)	35 (17.6)	23 (13.3)	65 (22.7)
Smoking status													
Never	674 (59.7)	110 (56.7)	116 (65.2)	143 (52.4)	305 (62.9)	121 (57.4)	105 (65.2)	131 (54.8)	317 (61.1)	674 (59.7)	124 (62.3)	102 (59.0)	162 (56.7)
Former	283 (25.0)	41 (21.1)	33 (18.5)	85 (31.1)	124 (25.6)	45 (21.3)	29 (18.0)	72 (30.1)	137 (26.4)	283 (25.0)	43 (21.6)	31 (17.9)	83 (29.0)
Current	173 (15.3)	43 (22.2)	29 (16.3)	45 (16.5)	56 (11.5)	45 (21.3)	27 (16.8)	36 (15.1)	65 (12.5)	173 (15.3)	32 (16.1)	40 (23.1)	41 (14.3)
Alcohol consumption at recruitment in g/day													
Non drinker	256 (22.6)	30 (15.4)	48 (27.0)	50 (18.3)	128 (26.4)	36 (17.0)	42 (26.1)	43 (18.0)	135 (26.0)	256 (22.6)	34 (17.1)	44 (25.4)	49 (17.2)
>0 to ≤3	384 (34.0)	58 (29.9)	60 (33.7)	92 (33.7)	174 (35.9)	63 (29.9)	55 (34.2)	81 (33.9)	185 (35.7)	384 (34.0)	62 (31.2)	56 (32.4)	111 (38.8)
>3 to ≤12	272 (24.1)	50 (25.8)	34 (19.1)	78 (28.6)	110 (22.6)	56 (26.5)	28 (17.4)	72 (30.1)	116 (22.3)	272 (24.1)	54 (27.1)	30 (17.3)	80 (28.0)
>12 to ≤24	137 (12.1)	35 (18.0)	23 (12.9)	27 (9.9)	52 (10.7)	36 (17.1)	22 (13.7)	24 (10.0)	55 (10.6)	137 (12.1)	29 (14.6)	29 (16.8)	31 (10.8)
>24	81 (7.2)	21 (10.8)	13 (7.3)	26 (9.5)	21 (4.3)	20 (9.5)	14 (8.7)	19 (8.0)	28 (5.4)	81 (7.2)	20 (10.0)	14 (8.1)	15 (5.2)
Age at menopause in years	49.02 (4.7)	48.25 (5.1)	49.80 (3.9)	48.85 (5.0)	49.13 (4.6)	48.68 (5.0)	49.39 (3.9)	48.83 (4.9)	49.12 (4.7)	49.02 (4.7)	48.83 (5.0)	49.17 (4.1)	48.62 (4.9)
Age at first menstrual period in years													
≤13	404 (35.7)	62 (32.0)	58 (32.6)	94 (34.4)	190 (39.2)	72 (34.1)	48 (29.8)	84 (35.2)	200 (38.5)	404 (35.7)	66 (33.2)	54 (31.2)	109 (38.1)
>13	726 (64.3)	132 (68.0)	120 (67.4)	179 (65.6)	295 (60.8)	139 (65.9)	113 (70.2)	155 (64.8)	319 (61.5)	726 (64.3)	133 (66.8)	119 (68.8)	177 (61.9)
Ever breastfed	825 (73.0)	145 (74.7)	127 (71.4)	197 (72.2)	356 (73.4)	162 (76.8)	110 (68.3)	174 (72.8)	379 (73.0)	825 (73.0)	149 (74.8)	123 (71.1)	208 (72.7)
Previous use of oral contraceptives at baseline	415 (36.7)	72 (37.1)	46 (25.8)	132 (48.4)	165 (34.0)	65 (30.8)	53 (32.9)	115 (48.1)	182 (35.1)	415 (36.7)	63 (31.7)	55 (31.8)	129 (45.1)
Previous use of menopausal hormone therapy at baseline	206 (18.2)	35 (18.0)	26 (14.6)	58 (21.3)	87 (17.9)	34 (16.1)	27 (16.8)	52 (21.8)	93 (17.9)	206 (18.2)	32 (16.1)	29 (16.8)	72 (25.2)
Number of full‐term pregnancies and age at first full‐term pregnancy													
Nulliparous	163 (14.4)	35 (18.0)	25 (14.0)	42 (15.4)	61 (12.5)	30 (14.2)	30 (18.6)	39 (16.3)	64 (12.3)	163 (14.4)	33 (16.6)	27 (15.6)	43 (15.0)
Age < 30 (1–2 children)	424 (37.5)	69 (35.6)	69 (38.8)	111 (40.7)	175 (36.1)	80 (37.9)	58 (36.1)	101 (42.3)	185 (35.6)	424 (37.5)	73 (36.7)	65 (37.6)	111 (38.8)
Age < 30 (≥3 children)	386 (34.2)	60 (30.9)	61 (34.3)	79 (28.9)	186 (38.4)	64 (30.3)	57 (35.4)	69 (28.9)	196 (37.8)	386 (34.2)	63 (31.6)	58 (33.5)	95 (33.2)
Age ≥ 30	157 (13.9)	30 (15.5)	23 (12.9)	41 (15.0)	63 (13.0)	37 (17.6)	16 (9.9)	30 (12.5)	74 (14.3)	157 (13.9)	30 (15.1)	23 (13.3)	37 (12.9)
Total energy intake in kcal/day	1885.0 (553.8)	1946.3 (531.2)	1829.1 (511.9)	1925.6 (440.5)	1858.2 (627.4)	1921.47 (503.3)	1849.25 (550.2)	1967.9 (646.6)	1843.1 (523.6)	1885.0 (553.8)	1884.0 (494.5)	1897.3 (558.6)	1898.8 (583.90)

^a^ Metabolically healthy/normal weight (BMI < 25 kg/m^2^ or Waist circumference < 80 cm or Waist‐to‐hip ratio < 0.8) plus within tertile 1 of C‐peptide.

^b^ Metabolically healthy/overweight (BMI ≥ 25 kg/m^2^ or Waist circumference ≥ 80 cm or Waist‐to‐hip ratio ≥ 0.8) plus within tertile 1 of C‐peptide.

^c^ Metabolically unhealthy/normal weight (BMI < 25 kg/m^2^ or Waist circumference < 80 cm or Waist‐to‐hip ratio < 0.8) plus above tertile 1 of C‐peptide.

^d^ Metabolically unhealthy/overweight (BMI ≥ 25 kg/m^2^ or Waist circumference ≥ 80 cm or Waist‐to‐hip ratio ≥ 0.8) plus above tertile 1 of C‐peptide.

* Values are mean (standard deviation).

### 3.2 Associations with breast cancer risk

Overweight and obese women had higher risks of postmenopausal breast cancer compared with normal weight women (OR = 1.50, 95% CI = 1.19–1.90 and OR = 1.37, 95% CI = 1.00–1.87, *p*_trend_ = 0.01, respectively; Table S1). High levels of WC were also associated with an increased risk of postmenopausal breast cancer (OR = 1.59, 95% CI = 1.16–2.17 for the highest quartile vs. the lowest, *p*_trend_ = 0.009), whereas high levels of WHR were not associated with the risk of postmenopausal breast cancer (OR = 1.14, 95% CI = 0.84–1.55 for the highest quartile vs. the lowest, *p*_trend_ = 0.43). In addition, circulating C‐peptide concentrations were positively and linearly associated with risk of postmenopausal breast cancer though the OR was only statistically significant (OR = 1.37, 95% CI = 1.00–1.87 for the highest tertile vs. the lowest, *p*_trend_ = 0.05).

### 3.3 Metabolic unhealthy/overweight or obese

Women classified as MUOW/OB had higher risk of postmenopausal breast cancer compared to MHNW women considering BMI (OR = 1.58, 95% CI = 1.14–2.19) and WC (OR = 1.51, 95% CI = 1.09–2.08) cut points and there was also a suggestive risk for the WHR (OR = 1.29, 95% CI = 0.94–1.77) definition (Table 3).

**TABLE 3:** Association between metabolic health–defined body size phenotypes using anthropometric cut points and breast cancer risk in postmenopausal women.

Metabolic health–defined body size phenotypes	Metabolically healthy		Metabolically unhealthy	
	Normal weight^a^	Overweight/Obesity^b^	Normal weight^c^	Overweight/Obesity^d^
Metabolic health/ BMI definition				
*N* cases/controls	85/194	88/178	124/273	313/485
Model 1 OR (95% CI)	1 [reference]	1.15 (0.80–1.66)	1.06 (0.75–1.50)	**1.50 (1.10–2.03)**
Model 2 OR (95% CI)	1 [reference]	1.17 (0.80–1.72)	1.02 (0.71–1.46)	**1.58 (1.14–2.19)**
Metabolic health/ WC definition				
*N* cases/controls	90/211	83/161	126/239	311/519
Model 1 OR (95% CI)	1 [reference]	1.28 (0.88–1.86)	1.28 (0.90–1.82)	**1.49 (1.10–2.03)**
Model 2 OR (95% CI)	1 [reference]	1.26 (0.85–1.85)	1.27 (0.88–1.83)	**1.51 (1.09–2.08)**
Metabolic health/WHR definition				
*N* cases/controls	96/199	77/173	154/286	283/472
Model 1 OR (95% CI)	1 [reference]	0.92 (0.63–1.34)	1.14 (0.82–1.59)	1.27 (0.94–1.72)
Model 2 OR (95% CI)	1 [reference]	0.92 (0.62–1.36)	1.14 (0.81–1.60)	1.29 (0.94–1.77)

*Note*: Model 1 was adjusted on matching criteria only and included age at blood collection, time of day at blood collection, and fasting status at blood collection. Model 2 was adjusted on matching factors, with additional adjustment for age at menarche, age at first full term pregnancy and parity, age at menopause, breastfeeding, ever use of contraceptive pills, ever use of menopausal hormonal therapy, physical activity index, alcohol consumption, smoking status, educational level, height, and energy intake. Bold values indicate statistically significant.

^a^ Metabolically healthy/normal weight (BMI < 25 kg/m^2^ or Waist circumference < 80 cm or Waist‐to‐hip ratio < 0.8) plus within tertile 1 of C‐peptide.

^b^ Metabolically healthy/overweight (BMI ≥ 25 kg/m^2^ or Waist circumference ≥ 80 cm or Waist‐to‐hip ratio ≥ 0.8) plus within tertile 1 of C‐peptide.

^c^ Metabolically unhealthy/normal weight (BMI < 25 kg/m^2^ or Waist circumference < 80 cm or Waist‐to‐hip ratio < 0.8) plus above tertile 1 of C‐peptide.

^d^ Metabolically unhealthy/overweight (BMI ≥ 25 kg/m^2^ or Waist circumference ≥ 80 cm or Waist‐to‐hip ratio ≥ 0.8) plus above tertile 1 of C‐peptide.

In stratified analyses, the positive association between MUOW/OB phenotype and postmenopausal breast cancer risk seemed to be stronger among non‐fasting women (OR_BMI_ = 2.11, 95% CI = 1.34–3.32, OR_WC_ = 2.15, 95% CI = 1.35–3.41 and OR_WHR_ = 1.93, 95% CI = 1.23–3.02), those who had previously used oral contraceptives (OR_BMI_ = 3.10, 95% CI = 1.19–8.06, OR_WC_ = 2.90, 95% CI = 1.03–8.16 and OR_WHR_ = 2.58, 95% CI = 0.99–6.86) and those aged 60 years or above at diagnosis (OR_BMI_ = 1.54, 95% CI = 1.03–2.30, OR_WC_ = 1.48, 95% CI = 1.00–2.21; Table S2). There was statistically significant heterogeneity observed across findings by fasting status (all *p*_heterogeneity_ < 0.05), whereas no evidence of heterogeneity was detected in findings according to ever use of oral contraceptives (all *p*_heterogeneity_ > 0.12) and age at diagnosis (all *p*_heterogeneity_ > 0.18) categories. In contrast, our stratified analyses by circulating levels of estradiol or estrone showed no statistically significant associations between MUOW/OB and postmenopausal breast cancer risk, except for among those with lower estrone levels and using the BMI definition (OR_BMI_ = 1.84, 95% CI = 1.04–3.28), with no heterogeneity detected in findings across categories (all *p*_heterogeneity_ > 0.26).

### 3.4 Metabolic unhealthy/normal weight

Women with MUNW were not at statistically significant elevated risk of postmenopausal breast cancer risk compared to MHNW women (OR_BMI_ = 1.02, 95% CI = 0.71–1.46, OR_WC_ = 1.27, 95% CI = 0.88–1.83 and OR_WHR_ = 1.14, 95% CI = 0.81–1.60; Table 3). We also observed no statistically significant associations between the MUNW phenotype and breast cancer risk in all subgroup analyses when considering BMI and WHR cut points (Table S2). However, when considering WC definition, the association was stronger and statistically significant among women who never used oral contraceptives (OR_WC_ = 1.81, 95% CI = 1.08–3.04) and not in those who had previously used oral contraceptives (OR_WC_ = 1.03, 95% CI = 0.38–2.77), although there was no heterogeneity detected in findings (*p*_heterogeneity_ = 0.32). There was also no heterogeneity in estimates across findings by circulating levels of estradiol and estrone (all *p*_heterogeneity_ > 0.10).

### 3.5 Metabolic healthy/overweight or obese

Women classified as MHOW/OB were not at elevated risk of postmenopausal breast cancer compared to MHNW women considering BMI (OR = 1.17, 95%CI = 0.80–1.72), WC (OR = 1.26, 95% CI = 0.85–1.85) and WHR (OR = 0.92, 95% CI = 0.62–1.36) cut points (Table 3). In addition, no statistically significant associations were found when comparing MHOW/OB phenotype to MHNW phenotype in all subgroups analyses (Table S2).

### 3.6 Sensitivity analyses

Exclusion of women who had a history of diabetes did not substantially change the magnitude of the associations, but the associations were attenuated and no longer statistically significant when excluding cases diagnosed within the first 2 years of follow‐up (Table S3). We also found no statistically significant associations when restricting the analyses to women who had never used MHT.

In sensitivity analyses considering only the upper tertile of C‐peptide concentration as the MU group, similar, and generally stronger associations were observed for the MUOW/OB group (Table S4). Using C‐peptide median cut points to define the metabolic health body size and shape phenotypes, we found that the positive associations were no longer statistically significant for MUOW/OB considering WC (OR = 1.27, 95% CI = 0.94–1.73) and WHR (OR = 1.12, 95% CI = 0.84–1.51), although the association remained statistically significant when considering BMI (OR = 1.41, 95% CI = 1.04–1.91). Defining women with C‐peptide levels above the first quartile as MU group, we found that the associations for MUOW/OB were no longer statistically significant. However, women classified as MHOW/OB had elevated risk compared to MHNW women when considering WC (OR = 2.12, 95% CI = 1.09–4.13; Table S4).

A similar pattern of results compared to those of the main analysis were observed when using a WC cut point of 88 cm, although the magnitude of the associations was attenuated for MUNW and MHOW/OB (Table S5). In contrast, women classified as MUNW were at elevated risk of postmenopausal breast cancer compared to MHNW when using a WHR cut point of 0.88.

## 4 DISCUSSION

In this prospective analysis, we found that women classified as MUOW/OB had elevated risk of postmenopausal breast cancer compared to MHNW women considering BMI, WC and WHR definitions. Our results also suggest that women classified as MHOW/OB or those with the MUNW phenotype were not at elevated risk of postmenopausal breast cancer compared to MHNW women.

Both adiposity and insulin resistance have been independently associated with higher postmenopausal breast cancer risk.^6^, ^7^, ^8^, ^9^, ^10^, ^14^ A large number of cohort studies have previously assessed the associations of metabolic phenotypes of obesity, defined by elevated levels of insulin or HOMA‐IR, with cardiovascular disease,^20^, ^21^, ^24^, ^25^, ^26^, ^44^, ^45^ diabetes,^18^, ^45^ and several cancer types.^19^, ^22^, ^30^, ^31^, ^32^, ^33^ Most of these previous studies reported higher risks of certain cancer types among MUNW or MUOW participants compared to MHNW participants, whereas lower risks were suggested among MHOW participants compared to MUOW.^30^, ^34^, ^45^

So far, four prospective studies have examined the association of BMI or WC, and insulin resistance/hyperinsulinemia in relation to breast cancer risk.^19^, ^35^, ^36^, ^37^ Using data from a case–cohort study (497 cases and 2830 cohort) within the Women's Health Initiative (WHI), Gunter and colleagues reported that overweight or obese and metabolically unhealthy participants, defined as having elevated HOMA‐IR, were at increased risk of postmenopausal breast cancer compared to MHNW participants, whereas MHOW participants did not have excess risk of the disease.^37^ Similarly, a subsequent analysis within the WHI (*n* ~ 21,000) found that overweight/obese women with metabolic abnormalities (defined as having ≥3 of the 5 parameters out of clinical range including central obesity, fasting glucose, blood pressure, and triglycerides) had a higher risk of postmenopausal breast cancer compared with normal weight women with no metabolic abnormalities.^36^ The association was stronger in women who had never used hormone therapy. However, normal weight women with metabolic abnormalities or those overweight with no metabolic abnormalities were not at elevated risk. In another analysis within the Framingham Heart Study (*n* = 3763), Moore and colleagues reported that overweight women with elevated blood glucose had a 2.6‐fold higher risk of reproductive cancers including a large proportion of postmenopausal breast cancers, whereas normal weight women with elevated glucose were not at higher risk.^19^ In this study, overweight women with normal glucose levels had a 1.7‐fold higher risk. In the Sister Study (*n* = 43,599), women classified as MUOW/OB, defined as being overweight or obese with one or more metabolic abnormalities, including central obesity, type 2 diabetes, dyslipidemia, and elevated blood pressure, had higher risk of developing postmenopausal breast cancer compared to normal weight women with no metabolic abnormalities and considering BMI, WC and WHR definitions.^35^ Consistent with these previous findings, we found that MUOW/OB women were at elevated risk of postmenopausal breast cancer compared to MHNW women and considering BMI, WC and WHR definitions.

A possible mechanism underlying the elevated risk of postmenopausal breast cancer among MUOW/OB women may implicate a direct effect of insulin which acts as a cancer promoter through its known mitotic and anti‐apoptotic activities.^11^, ^12^ Experimental studies suggest that insulin plays a role in cancer initiation and progression by increasing the likelihood of genetic alterations in normal cells and malignant cells.^46^ Elevated circulating levels of insulin could also influence breast cancer risk by regulating sex hormone synthesis and chronic inflammation which in turn increase the risk of the disease.^6^, ^16^, ^17^ A recent study reported that adults with MUOW phenotype (defined using metabolic syndrome definition with BMI) had higher levels of serum concentrations of inflammatory cytokines, such as interleukin (IL‐1β), IL‐6, IL‐8, IL‐10, and tumor necrosis factor‐α, compared to adults with MHNW,^47^ which suggests that excess body fat and altered metabolic profile are also linked to inflammation. Another potential hypothesis to explain the observed associations is related to other factors influencing the metabolic dysfunction. Hypothetically, an unhealthy diet and sedentary behavior may influence the development of metabolic dysfunction,^48^ thereby increasing the risk of breast cancer. Indeed, previous studies reported that participants with metabolic dysfunction tend to report lower intakes of plant‐based diets including fruits, vegetables, and wholegrains, to have higher intakes of sugar and saturated fat, and to report higher levels of sedentary behavior compared to metabolically healthy participants.^49^, ^50^ However, associations remained statistically significant despite adjustment for physical activity index and total energy from the diet.

Our findings suggest that women with MHOW/OB or those with MUNW did not have a significantly increased risk of postmenopausal breast cancer compared to MHNW women. These findings lend support to those from two previous studies reporting no statistically significant association among normal weight women with metabolic abnormalities,^35^, ^37^ but are inconsistent with two other previous studies showing an excess breast cancer risk among overweight or obese women without metabolic abnormalities.^19^, ^36^ However, in our study, the non‐statistically significant association among normal weight with elevated insulin levels or among overweight and obese women with normal insulin levels could be due to the lower sample size in these subgroups and hence lack of statistical power. Given that C‐peptide levels seemed to be higher among MUOW/OB women (5.23 ng/mL) compared to MUNW women (4.75 ng/mL), it could also be possible that a much higher insulin level is required to increase breast cancer risk. In a recent investigation within the EPIC cohort, elevated risk of endometrial cancer was observed among women classified as MUNW, MUOW, and MHOW phenotypes compared with MHNW.^34^ Similarly, a study with the EPIC cohort reported that normal‐weight women with elevated insulin levels had at higher colorectal cancer risk compared to those of normal‐weight with normal insulin levels,^30^ whereas a lower colorectal cancer risk was found among the overweight women with normal insulin levels. However, we did not find a lower risk of postmenopausal breast cancer among overweight women with normal insulin levels. We could hypothesize that biological mechanisms involving adiposity and insulin may differ between colorectal and breast cancers or this could be related to sample size. However, further studies with larger sample sizes are needed to investigate these associations and explore the potential biological mechanisms linking insulin and other biological markers with breast cancer risk.

Our results indicate that the elevated risk among MUOW/OB phenotype appeared to be stronger in women who had ever used oral contraceptives, compared to never users, suggesting that sex steroids hormone may influence these associations. When further excluding women who had ever used MHT, the associations were no longer statistically significant with no heterogeneity detected in findings across categories. In addition, we found that the positive associations were restricted to non‐fasting women with evidence of significant heterogeneity across categories of fasting status. Most of the previous studies considered fasting glucose/insulin or fasting biomarkers. Only the analyses from Framingham study considered fasting and non‐fasting samples; and the authors reported that non‐fasting samples also could be used to identify cases of diabetes with sensitivity equal to that of fasting samples.^19^ However, since our study is the first to conduct subgroup analysis by fasting status, further studies are needed to better understand the elevated risk among non‐fasting women. In addition, exclusion of participants with diabetes did not substantially impact our results. When excluding cases diagnosed within the first 2 years of follow‐up, the positive associations for MUOW/OB were attenuated, and the relationship did not reach statistical significance. This could be explained by a possible reverse causality in the associations, or by the loss of power due to the exclusion of 209 cases and their matched controls.

Our study has some limitations that should be taken into consideration when interpreting these findings. First, a single measurement of C‐peptide with a study‐specific cut point was used to define metabolic health categories which may not reflect a complete and objective definition of metabolic healthy status. Therefore, some degree of misclassification cannot be excluded. However, although similar patterns of results compared to those of the main analysis were obtained when considering the first quartile or median of C‐peptide as the metabolically healthy group, the associations were no longer statistically significant—probably due to the small sample size in some categories. In addition, although previous studies have used different markers such as glucose or insulin levels, HOMA‐IR, and the MetS to define “metabolic health”, two recent cohort studies have used concentration of C‐peptide as a marker of metabolic health status since C‐peptide has been reported to be a good indicator for long‐term insulin secretion.^30^, ^34^ A recent review supports that adiposity with elevated levels of a single biomarker, including HOMA‐IR, C‐peptide, or fasting insulin could also be used to define metabolic health phenotype.^51^ The authors suggest that being metabolically unhealthy as the presence of at least one, two, or three MetS criteria, regardless of BMI category, is associated with a higher risk of at least four cancer types. Moreover, in several studies, similar results were reported when a single marker was used to define metabolic health compared with the use of MetS.^36^, ^37^, ^52^ Another limitation of the study includes the small sample size in some subgroup analyses, and the incomplete information on breast cancer subtypes. The observed associations may vary according to breast cancer receptor subtype since a recent study reported a strong positive association between MUOW/OB phenotype and estrogen receptor‐positive breast cancer risk.^35^ However, more research with detailed information on breast cancer subtype is required to explore this hypothesis. Despite these limitations, this study has several strengths, including its prospective design, the detailed information on potential risk factors for breast cancer from seven countries, and the use of C‐peptide, a validated marker of hyperinsulinemia,^53^, ^54^, ^55^ to define metabolic heath status. In addition, breast cancer cases were confirmed through a combination of several methods including cancer registries, health insurance records and active follow‐up.

In conclusion, our findings suggest that being overweight or obese and metabolically unhealthy is associated with an increased risk of postmenopausal breast cancer compared to metabolically healthy normal weight women and considering BMI, WC and WHR measurements as markers of adiposity and C‐peptide as marker of metabolic health. Overweight or obese women with normal insulin levels are not at higher risk of breast cancer. Although additional research is required, these findings highlighted the importance of assessing the effects of anthropometric measures in conjunction with metabolic parameters to identify women at higher risk of breast cancer.

## AUTHOR CONTRIBUTIONS

**Yahya Mahamat‐Saleh:** Conceptualization (equal); data curation (equal); formal analysis (equal); funding acquisition (equal); investigation (equal); methodology (equal); resources (equal); software (equal); visualization (equal); writing – original draft (equal); writing – review and editing (equal). **Sabina Rinaldi:** Conceptualization (equal); data curation (equal); funding acquisition (equal); methodology (equal); resources (equal); supervision (equal); writing – review and editing (equal). **Rudolf Kaaks:** Investigation (equal); methodology (equal); resources (equal); writing – review and editing (equal). **Carine Biessy:** Data curation (equal); formal analysis (equal); methodology (equal); project administration (equal); visualization (equal); writing – review and editing (equal). **Esther Gonzalez Gil:** Investigation (equal); methodology (equal); resources (equal); writing – review and editing (equal). **Neil Murphy:** Investigation (equal); methodology (equal); resources (equal); writing – review and editing (equal). **Charlotte Le Cornet:** Investigation (equal); methodology (equal); resources (equal); writing – review and editing (equal). **Jose Maria Huerta:** Investigation (equal); methodology (equal); resources (equal); writing – review and editing (equal). **Sabina Sieri:** Investigation (equal); methodology (equal); resources (equal); writing – review and editing (equal). **Anne Tjonneland:** Investigation (equal); methodology (equal); resources (equal); writing – review and editing (equal). **Lene Mellemkjaer:** Investigation (equal); methodology (equal); resources (equal); writing – review and editing (equal). **Marcela Guevara:** Investigation (equal); methodology (equal); resources (equal); writing – review and editing (equal). **Kim Overvad:** Investigation (equal); methodology (equal); resources (equal); writing – review and editing (equal). **Aurora Perez‐Cornago:** Investigation (equal); methodology (equal); resources (equal); writing – review and editing (equal). **Sandar Tin Tin:** Investigation (equal); methodology (equal); resources (equal); writing – review and editing (equal). **Lisa Padroni:** Investigation (equal); methodology (equal); resources (equal); writing – review and editing (equal). **Vittorio Simeon:** Investigation (equal); methodology (equal); resources (equal); writing – review and editing (equal). **Giovanna Masala:** Investigation (equal); methodology (equal); resources (equal); writing – review and editing (equal). **Anne May:** Investigation (equal); methodology (equal); resources (equal); writing – review and editing (equal). **E. M. Monninkhof:** Investigation (equal); methodology (equal); resources (equal); writing – review and editing (equal). **Sofia Christakoudi:** Investigation (equal); methodology (equal); resources (equal); writing – review and editing (equal). **Alicia K. Heath:** Investigation (equal); methodology (equal); resources (equal); writing – review and editing (equal). **Konstantinos K. Tsilidis:** Investigation (equal); methodology (equal); resources (equal); writing – review and editing (equal). **Antonio Agudo:** Investigation (equal); methodology (equal); resources (equal); writing – review and editing (equal). **Matthias B. Schulze:** Investigation (equal); methodology (equal); resources (equal); writing – review and editing (equal). **Joseph Rothwell:** Investigation (equal); methodology (equal); resources (equal); writing – review and editing (equal). **Claire Cadeau:** Investigation (equal); methodology (equal); resources (equal); writing – review and editing (equal). **Gianluca Severi:** Investigation (equal); methodology (equal); resources (equal); writing – review and editing (equal). **Elisabete Weiderpass:** Investigation (equal); methodology (equal); resources (equal); writing – review and editing (equal). **Marc Gunter:** Conceptualization (equal); funding acquisition (equal); investigation (equal); methodology (equal); resources (equal); supervision (equal); writing – review and editing (equal). **Laure Dossus:** Conceptualization (equal); data curation (equal); funding acquisition (equal); investigation (equal); methodology (equal); resources (equal); supervision (equal); writing – original draft (equal); writing – review and editing (equal).

## FUNDING INFORMATION

Yahya Mahamat‐Saleh is a postdoctoral scientist at the International Agency for Research on Cancer and supported by the Fondation ARC pour la recherche sur le cancer ARCPOST‐DOC2021080004105. The coordination of EPIC is financially supported by International Agency for Research on Cancer (IARC) and also by the Department of Epidemiology and Biostatistics, School of Public Health, Imperial College London which has additional infrastructure support provided by the NIHR Imperial Biomedical Research Centre (BRC). The national cohorts are supported by: Danish Cancer Society (Denmark); Ligue Contre le Cancer, Institut Gustave Roussy, Mutuelle Générale de l'Education Nationale, Institut National de la Santé et de la Recherche Médicale (INSERM; France); German Cancer Aid, German Cancer Research Center (DKFZ), German Institute of Human Nutrition Potsdam‐Rehbruecke (DIfE), Federal Ministry of Education and Research (BMBF; Germany); Associazione Italiana per la Ricerca sul Cancro‐AIRC‐Italy, Compagnia di SanPaolo and National Research Council (Italy); Dutch Ministry of Public Health, Welfare and Sports (VWS), Netherlands Cancer Registry (NKR), LK Research Funds, Dutch Prevention Funds, Dutch ZON (Zorg Onderzoek Nederland), World Cancer Research Fund (WCRF), Statistics Netherlands (The Netherlands); Health Research Fund (FIS)—Instituto de Salud Carlos III (ISCIII), Regional Governments of Andalucía, Asturias, Basque Country, Murcia and Navarra, and the Catalan Institute of Oncology—ICO (Spain); Swedish Cancer Society, Swedish Research Council and County Councils of Skåne and Västerbotten (Sweden); Cancer Research UK (14,136 to EPIC‐Norfolk; C8221/A29017 to EPIC‐Oxford), Medical Research Council (1,000,143 to EPIC‐Norfolk; MR/M012190/1 to EPIC‐Oxford; United Kingdom).

## CONFLICT OF INTEREST STATEMENT

The authors have no conflict of interest to disclose.

## ETHICS APPROVAL AND CONSENT TO PARTICIPATE

The EPIC study was conducted in accordance with the Declaration of Helsinki. The study was approved by the local ethical committees in participating countries and the IARC ethical committee. All participants provided written informed consent for data collection and storage, as well as individual follow‐up before study entry. This study is listed at clinicaltrials.gov as NCT03285230.

## CONSENT FOR PUBLICATION

Not applicable.

## IARC DISCLAIMER

Where authors are identified as personnel of the International Agency for Research on Cancer / World Health Organization, the authors alone are responsible for the views expressed in this article and they do not necessarily represent the decisions, policy, or views of the International Agency for Research on Cancer/World Health Organization.

## Supporting information


Data S1

## Data Availability

For information on how to submit an application for gaining access to EPIC data and/or biospecimens, please follow the instructions at https://login.research4life.org/tacsgr0epic_iarc_fr/access/index.php.
