# Supplementary material for: Metabolically defined body size and body shape phenotypes and risk of postmenopausal breast cancer in the European Prospective Investigation into Cancer and Nutrition
Source: Cancer Med. 2023 Apr 25;12(11):12668–82. doi: 10.1002/cam4.5896 (PMC10278526; doi:10.1002/cam4.5896)
Supplement: Supplementary file 1 — Data S1 [file CAM4-12-12668-s001.docx]

| **Table S1: Associations between body mass index, waist circumference, waist-to-hip ratio or serum C-peptide and breast cancer risk in postmenopausal women** | | | | |
| --- | --- | --- | --- | --- |
|  | Cut off points | N cases/controls | Model 1  OR (95% CI) | Model 2  OR (95% CI) |
| **Body Mass Index (kg/m^2^)** | |  |  |  |
| Underweight | <18.5 | 4/7 | 1.16 (0.34-3.99) | 1.32 (0.37-4.72) |
| Normal weight | 18.5-24.99 | 205/460 | 1 [reference] | 1 [reference] |
| Overweight | 25.00-29.99 | 287/451 | **1.43 (1.14-1.79)** | **1.50 (1.19-1.90)** |
| Obesity | ≥30.00 | 114/212 | 1.22 (0.91-1.63) | **1.37 (1.00-1.87)** |
| P_-trend_ |  |  | **0.05** | **0.01** |
| **Waist circumference (cm)** | |  |  |  |
| Quartile 1 | <75.99 | 123/288 | 1 [reference] | 1 [reference] |
| Quartile 2 | 76.00-82.99 | 166/291 | **1.36 (1.02-1.81)** | **1.35 (1.01-1.82)** |
| Quartile 3 | 83.00-89.99 | 147/279 | 1.27 (0.94-1.70) | 1.27 (0.93-1.74) |
| Quartile 4 | ≥90.00 | 174/272 | **1.54 (1.14-2.08)** | **1.59 (1.16-2.17)** |
| P_-trend_ |  |  | **0.01** | **0.009** |
| **Waist-to-hip ratio** |  |  |  |  |
| Quartile 1 | <0.76 | 145/279 | 1 [reference] | 1 [reference] |
| Quartile 2 | 0.77-0.80 | 143/271 | 1.00 (0.75-1.34) | 1.01 (0.75-1.37) |
| Quartile 3 | 0.81-0.84 | 151/286 | 1.00 (0.75-1.34) | 1.01 (0.75-1.36) |
| Quartile 4 | ≥0.85 | 171/294 | 1.12 (0.83-1.50) | 1.14 (0.84-1.55) |
| P_-trend_ |  |  | 0.49 | 0.43 |
| **Concentrations of C-peptide (ng/ml)** | | |  |  |
| Tertile 1 | <2.76 | 173/372 | 1 [reference] | 1 [reference] |
| Tertile 2 | 2.76-4.22 | 202/373 | 1.20 (0.93-1.55) | 1.17 (0.89-1.54) |
| Tertile 3 | ≥4.23 | 235/385 | **1.40 (1.05-1.87)** | **1.37 (1.00-1.87)** |
| P_-trend_ |  |  | **0.02** | **0.05** |

Model 1 was adjusted on matching criteria only and included age at blood collection, time of day at blood collection and fasting status at blood collection.

Model 2 was adjusted on matching factors, with additional adjustment for age at menarche, age at first full term pregnancy and parity, age at menopause, breastfeeding, ever use of contraceptive pills, ever use of menopausal hormonal therapy, physical activity index, alcohol consumption, smoking status, educational level, height, and energy intake.

| **Table S2: Association between metabolic health–defined body size phenotypes using anthropometric cut-points and breast cancer risk in postmenopausal women according to fasting status at blood collection, ever use of oral contraceptives, estrone, and estradiol levels, and age at diagnosis** | | | | | | | | | | | | |
| --- | --- | --- | --- | --- | --- | --- | --- | --- | --- | --- | --- | --- |
|  | **Metabolic Health/BMI Definition** | | | | **Metabolic Health/WC Definition** | | | | **Metabolic Health/WHR Definition** | | | |
|  | **MHNW^1^** | **MHOW/OB^2^** | **MUNW^3^** | **MUOW/OB^4^** | **MHNW^1^** | **MHOW/OB^2^** | **MUNW^3^** | **MUOW/OB^4^** | **MHNW^1^** | **MHOW/OB^2^** | **MUNW^3^** | **MUOW/OB^4^** |
| **Fasting status at blood collection** | |  |  |  |  |  |  |  |  |  |  |  |
| *Nonfasting* |  |  |  |  |  |  |  |  |  |  |  |  |
| N cases/controls | 40/101 | 27/82 | 109/244 | 242/345 | 38/106 | 29/77 | 107/208 | 244/381 | 41/107 | 26/76 | 136/253 | 215/336 |
| Model 1 | 1 [reference] | 0.84 (0.48-1.48) | 1.20 (0.77-1.87) | **1.87 (1.22-2.87)** | 1 [reference] | 1.13 (0.64-1.98) | 1.53 (0.96-2.44) | **1.94 (1.26-3.01)** | 1 [reference] | 0.91 (0.50-1.66) | 1.47 (0.95-2.28) | **1.78 (1.15-2.73)** |
| Model 2 | 1 [reference] | 0.90 (0.50-1.62) | 1.21 (0.76-1.92) | **2.11 (1.34-3.32)** | 1 [reference] | 1.21 (0.67-2.19) | 1.57 (0.96-2.55) | **2.15 (1.35-3.41)** | 1 [reference] | 0.97 (0.52-1.80) | 1.53 (0.97-2.42) | **1.93 (1.23-3.02)** |
| *Fasting* |  |  |  |  |  |  |  |  |  |  |  |  |
| N cases/controls | 45/93 | 61/96 | 15/29 | 71/140 | 52/105 | 54/84 | 19/31 | 67/138 | 55/92 | 51/97 | 18/33 | 68/136 |
| Model 1 | 1 [reference] | 1.40 (0.85-2.31) | 1.03 (0.50-2.13) | 1.09 (0.69-1.72) | 1 [reference] | 1.37 (0.83-2.27) | 1.16 (0.60-2.24) | 1.03 (0.65-1.62) | 1 [reference] | 0.85 (0.52-1.40) | 0.87 (0.45-1.67) | 0.82 (0.52-1.29) |
| Model 2 | 1 [reference] | 1.32 (0.76-2.29) | 1.06 (0.48-2.34) | 1.03 (0.62-1.71) | 1 [reference] | 1.18 (0.68-2.05) | 1.32 (0.64-2.72) | 0.90 (0.54-1.48) | 1 [reference] | 0.81 (0.47-1.40) | 1.02 (0.49-2.11) | 0.75 (0.46-1.24) |
| **Oral contraceptive use** |  |  |  |  |  |  |  |  |  |  |  |  |
| *Never* |  |  |  |  |  |  |  |  |  |  |  |  |
| N cases/controls | 62/122 | 71/132 | 82/141 | 205/321 | 66/146 | 67/108 | 84/124 | 203/338 | 70/136 | 63/118 | 105/157 | 182/305 |
| Model 1 | 1 [reference] | 1.07 (0.67-1.71) | 1.11 (0.68-1.82) | 1.23 (0.82-1.86) | 1 [reference] | 1.59 (0.99-2.54) | **1.78 (1.09-2.92)** | 1.38 (0.92-2.06) | 1 [reference] | 0.95 (0.59-1.53) | 1.29 (0.82-2.02) | 1.06 (0.70-1.59) |
| Model 2 | 1 [reference] | 1.17 (0.71-1.92) | 1.07 (0.64-1.81) | 1.39 (0.90-2.15) | 1 [reference] | 1.58 (0.96-2.58) | **1.81 (1.08-3.04)** | 1.42 (0.93-2.17) | 1 [reference] | 0.93 (0.56-1.54) | 1.33 (0.83-2.13) | 1.07 (0.70-1.66) |
| *Ever* |  |  |  |  |  |  |  |  |  |  |  |  |
| N cases/controls | 23/72 | 17/46 | 42/132 | 108/164 | 24/65 | 16/53 | 42/115 | 108/181 | 26/63 | 14/55 | 49/129 | 101/167 |
| Model 1 | 1 [reference] | 1.27 (0.46-3.54) | 0.95 (0.46-1.97) | **2.56 (1.24-5.30)** | 1 [reference] | 1.24 (0.45-3.45) | 1.06 (0.50-2.25) | **2.35 (1.09-5.07)** | 1 [reference] | 1.16 (0.40-3.33) | 1.07 (0.52-2.22) | **2.29 (1.08-4.87)** |
| Model 2 | 1 [reference] | 1.27 (0.36-4.50) | 0.90 (0.35-2.34) | **3.10 (1.19-8.06)** | 1 [reference] | 1.45 (0.41-5.16) | 1.03 (0.38-2.77) | **2.90 (1.03-8.16)** | 1 [reference] | 1.36 (0.38-4.84) | 1.05 (0.40-2.77) | 2.58 (0.99-6.86) |
| **Estrone levels** |  |  |  |  |  |  |  |  |  |  |  |  |
| *<150.8 pmol/l* |  |  |  |  |  |  |  |  |  |  |  |  |
| N cases/controls | 45/105 | 35/89 | 60/158 | 127/207 | 49/111 | 31/83 | 68/139 | 119/226 | 51/108 | 29/86 | 82/159 | 105/206 |
| Model 1 | 1 [reference] | 1.02 (0.55-1.86) | 1.16 (0.65-2.07) | 1.66 (0.98-2.81) | 1 [reference] | 0.95 (0.49-1.83) | 1.46 (0.80-2.67) | 1.41 (0.81-2.44) | 1 [reference] | 0.65 (0.34-1.26) | 1.34 (0.76-2.36) | 1.14 (0.67-1.96) |
| Model 2 | 1 [reference] | 0.91 (0.47-1.77) | 1.17 (0.63-2.19) | **1.84 (1.04-3.28)** | 1 [reference] | 0.74 (0.35-1.53) | 1.59 (0.83-3.06) | 1.34 (0.73-2.45) | 1 [reference] | 0.67 (0.33-1.37) | 1.51 (0.82-2.79) | 1.33 (0.74-2.38) |
| *≥150.8 pmol/l* |  |  |  |  |  |  |  |  |  |  |  |  |
| N cases/controls | 35/80 | 46/83 | 59/104 | 164/258 | 37/90 | 44/73 | 51/88 | 172/274 | 40/83 | 41/80 | 61/114 | 162/248 |
| Model 1 | 1 [reference] | 1.03 (0.53-1.98) | 1.24 (0.65-2.37) | 1.48 (0.85-2.60) | 1 [reference] | 1.28 (0.66-2.48) | 1.36 (0.68-2.71) | 1.66 (0.95-2.88) | 1 [reference] | 0.98 (0.51-1.87) | 1.08 (0.57-2.04) | 1.51 (0.88-2.58) |
| Model 2 | 1 [reference] | 0.98 (0.48-2.03) | 1.26 (0.62-2.55) | 1.56 (0.84-2.90) | 1 [reference] | 1.25 (0.59-2.64) | 1.28 (0.61-2.71) | 1.78 (0.97-3.27) | 1 [reference] | 1.08 (0.52-2.23) | 1.22 (0.62-2.41) | 1.68 (0.94-3.00) |
| **Estradiol levels** |  |  |  |  |  |  |  |  |  |  |  |  |
| *<88.50 pmol/l* |  |  |  |  |  |  |  |  |  |  |  |  |
| N cases/controls | 43/118 | 45/91 | 59/155 | 108/242 | 49/125 | 39/84 | 63/152 | 104/245 | 52/113 | 36/96 | 63/167 | 104/230 |
| Model 1 | 1 [reference] | 1.34 (0.76-2.36) | 0.94 (0.51-1.75) | 1.15 (0.68-1.93) | 1 [reference] | 1.38 (0.75-2.52) | 1.11 (0.60-2.05) | 1.10 (0.65-1.86) | 1 [reference] | 0.86 (0.46-1.61) | 0.89 (0.48-1.63) | 0.90 (0.53-1.51) |
| Model 2 | 1 [reference] | 1.31 (0.71-2.40) | 0.93 (0.48-1.80) | 1.15 (0.66-2.01) | 1 [reference] | 1.27 (0.66-2.44) | 1.11 (0.58-2.13) | 1.06 (0.60-1.88) | 1 [reference] | 0.90 (0.46-1.73) | 0.88 (0.46-1.69) | 0.92 (0.53-1.62) |
| *≥88.50 pmol/l* |  |  |  |  |  |  |  |  |  |  |  |  |
| N cases/controls | 42/74 | 42/82 | 63/117 | 201/241 | 41/84 | 43/72 | 61/86 | 203/272 | 44/83 | 40/73 | 88/116 | 176/242 |
| Model 1 | 1 [reference] | 1.03 (0.53-1.98) | 1.24 (0.65-2.37) | 1.48 (0.85-2.60) | 1 [reference] | 1.37 (0.71-2.63) | 1.74 (0.94-3.22) | **1.84 (1.09-3.11)** | 1 [reference] | 1.11 (0.58-2.14) | 1.92 (1.08-3.40) | 1.53 (0.91-2.58) |
| Model 2 | 1 [reference] | 0.98 (0.48-2.03) | 1.26 (0.62-2.55) | 1.56 (0.84-2.90) | 1 [reference] | 1.28 (0.63-2.60) | 1.78 (0.92-3.46) | 1.68 (0.95-2.98) | 1 [reference] | 1.03 (0.51-2.08) | 1.88 (1.02-3.48) | 1.40 (0.80-2.46) |
| **Age at diagnosis** |  |  |  |  |  |  |  |  |  |  |  |  |
| *<60 years* |  |  |  |  |  |  |  |  |  |  |  |  |
| N cases/controls | 31/66 | 23/51 | 29/72 | 83/117 | 32/69 | 22/48 | 32/66 | 80/123 | 32/70 | 22/47 | 34/77 | 78/112 |
| Model 1 | 1 [reference] | 1.00 (0.51-1.96) | 0.79 (0.41-1.53) | 1.45 (0.86-2.45) | 1 [reference] | 1.03 (0.52-2.01) | 1.02 (0.54-1.93) | 1.38 (0.81-2.34) | 1 [reference] | 1.05 (0.53-2.09) | 0.92 (0.50-1.70) | 1.49 (0.88-2.54) |
| Model 2 | 1 [reference] | 1.06 (0.49-2.28) | 0.78 (0.38-1.59) | 1.80 (0.99-3.28) | 1 [reference] | 1.13 (0.54-2.36) | 1.06 (0.53-2.14) | 1.67 (0.93-3.02) | 1 [reference] | 1.24 (0.58-2.64) | 1.08 (0.56-2.07) | **1.85 (1.02-3.33)** |
| *≥60 years* |  |  |  |  |  |  |  |  |  |  |  |  |
| N cases/controls | 54/128 | 65/127 | 95/201 | 230/368 | 58/142 | 61/113 | 94/173 | 231/396 | 64/129 | 55/126 | 120/209 | 205/360 |
| Model 1 | 1 [reference] | 1.23 (0.79-1.91) | 1.17 (0.77-1.78) | **1.53 (1.05-2.23)** | 1 [reference] | 1.42 (0.91-2.23) | 1.42 (0.93-2.16) | **1.55 (1.07-2.27)** | 1 [reference] | 0.87 (0.55-1.38) | 1.21 (0.81-1.80) | 1.18 (0.82-1.72) |
| Model 2 | 1 [reference] | 1.19 (0.75-1.88) | 1.14 (0.74-1.77) | **1.54 (1.03-2.30)** | 1 [reference] | 1.29 (0.81-2.07) | 1.40 (0.90-2.17) | **1.48 (1.00-2.21)** | 1 [reference] | 0.81 (0.51-1.31) | 1.17 (0.77-1.78) | 1.15 (0.78-1.70) |

Model 1 was adjusted on matching criteria only and included age at blood collection, time of day at blood collection and fasting status at blood collection.

Model 2 was adjusted on matching factors, with additional adjustment for age at menarche, age at first full term pregnancy and parity, age at menopause, breastfeeding, ever use of contraceptive pills, ever use of menopausal hormonal therapy, physical activity index, alcohol consumption, smoking status, educational level, height, and energy intake.

^1^Metabolically healthy/normal weight (BMI < 25 kg/m^2^ or Waist circumference <80 cm or Waist-to-hip ratio <0.8) plus within tertile 1 of C-peptide.

^2^Metabolically healthy/overweight or obese (BMI ≥ 25 kg/m^2^ or Waist circumference ≥80 cm or Waist-to-hip ratio ≥0.8) plus within tertile 1 of C-peptide.

^3^Metabolically unhealthy/normal weight (BMI < 25 kg/m^2^ or Waist circumference <80cm or Waist-to-hip ratio <0.8) plus above tertile 1 of C-peptide.

^4^Metabolically unhealthy/overweight or obese (BMI ≥ 25 kg/m^2^ or Waist circumference ≥80cm or Waist-to-hip ratio ≥0.8) plus above tertile 1 of C-peptide

| **Table S3: Association between metabolic health–defined body size phenotypes and breast cancer risk in postmenopausal women when** **considering women without diabetes only, excluding cases diagnosed within the first 2 y of follow-up and their matched controls or women who had ever used HRT** | | | | |
| --- | --- | --- | --- | --- |
| Metabolic health–defined body size phenotypes | **Metabolically healthy** | | **Metabolically unhealthy** | |
|  | **Normal weight^1^** | **Overweight/Obesity^2^** | **Normal weight^3^** | **Overweight/Obesity^4^** |
| ***Women with no history of diabetes only*** |  |  |  |  |
| **Metabolic Health/ BMI Definition** |  |  |  |  |
| N cases/controls | 84/191 | 83/172 | 123/268 | 300/458 |
| Model 1 OR (95% CI) | 1 [reference] | 1.09 (0.75-1.59) | 1.05 (0.74-1.50) | **1.51 (1.10-2.06)** |
| Model 2 OR (95% CI) | 1 [reference] | 1.09 (0.74-1.62) | 1.02 (0.71-1.48) | **1.58 (1.13-2.20)** |
| **Metabolic Health/ WC Definition** |  |  |  |  |
| N cases/controls | 89/209 | 78/154 | 124/236 | 299/490 |
| Model 1 OR (95% CI) | 1 [reference] | 1.22 (0.83-1.79) | 1.26 (0.88-1.79) | **1.51 (1.10-2.06)** |
| Model 2 OR (95% CI) | 1 [reference] | 1.17 (0.79-1.75) | 1.25 (0.86-1.81) | **1.52 (1.09-2.11)** |
| **Metabolic Health/ WHR Definition** |  |  |  |  |
| N cases/controls | 94/199 | 73/164 | 151/285 | 272/441 |
| Model 1 OR (95% CI) | 1 [reference] | 0.92 (0.62-1.36) | 1.14 (0.82-1.60) | 1.33 (0.98-1.82) |
| Model 2 OR (95% CI) | 1 [reference] | 0.89 (0.59-1.33) | 1.14 (0.81-1.62) | 1.34 (0.97-1.85) |
| ***Excluding cases diagnosed within the first 2 y of follow-up*** |  |  |  |  |
| **Metabolic Health/ BMI Definition** |  |  |  |  |
| N cases/controls | 52/113 | 52/105 | 90/182 | 207/346 |
| Model 1 OR (95% CI) | 1 [reference] | 1.07 (0.67-1.71) | 1.07 (0.70-1.63) | 1.29 (0.88-1.89) |
| Model 2 OR (95% CI) | 1 [reference] | 1.05 (0.65-1.71) | 1.04 (0.67-1.62) | 1.30 (0.87-1.94) |
| **Metabolic Health/ WC Definition** |  |  |  |  |
| N cases/controls | 57/122 | 47/96 | 89/168 | 208/360 |
| Model 1 OR (95% CI) | 1 [reference] | 1.08 (0.67-1.75) | 1.13 (0.74-1.73) | 1.26 (0.87-1.84) |
| Model 2 OR (95% CI) | 1 [reference] | 1.03 (0.62-1.71) | 1.11 (0.72-1.73) | 1.25 (0.84-1.85) |
| **Metabolic Health/ WHR Definition** |  |  |  |  |
| N cases/controls | 58/118 | 46/100 | 108/207 | 189/321 |
| Model 1 OR (95% CI) | 1 [reference] | 0.91 (0.56-1.49) | 1.05 (0.70-1.57) | 1.19 (0.81-1.73) |
| Model 2 OR (95% CI) | 1 [reference] | 0.91 (0.55-1.52) | 1.03 (0.68-1.57) | 1.21 (0.81-1.78) |
| ***Women who never use HRT only*** |  |  |  |  |
| **Metabolic Health/ BMI Definition** |  |  |  |  |
| N cases/controls | 61/122 | 71/132 | 82/141 | 205/321 |
| Model 1 OR (95% CI) | 1 [reference] | 1.14 (0.75-1.71) | 0.88 (0.59-1.32) | 1.28 (0.90-1.83) |
| Model 2 OR (95% CI) | 1 [reference] | 1.17 (0.76-1.79) | 0.86 (0.56-1.30) | 1.35 (0.92-1.97) |
| **Metabolic Health/ WC Definition** |  |  |  |  |
| N cases/controls | 66/146 | 67/108 | 84/124 | 203/338 |
| Model 1 OR (95% CI) | 1 [reference] | 1.21 (0.80-1.84) | 1.08 (0.72-1.60) | 1.23 (0.87-1.74) |
| Model 2 OR (95% CI) | 1 [reference] | 1.24 (0.81-1.91) | 1.07 (0.71-1.63) | 1.26 (0.87-1.81) |
| **Metabolic Health/ WHR Definition** |  |  |  |  |
| N cases/controls | 70/136 | 63/118 | 105/157 | 182/305 |
| Model 1 OR (95% CI) | 1 [reference] | 0.94 (0.62-1.42) | 1.08 (0.74-1.59) | 1.04 (0.74-1.47) |
| Model 2 OR (95% CI) | 1 [reference] | 0.92 (0.60-1.43) | 1.08 (0.72-1.60) | 1.04 (0.73-1.49) |

Model 1 was adjusted on matching criteria only and included age at blood collection, time of day at blood collection and fasting status at blood collection.

Model 2 was adjusted on matching factors, with additional adjustment for age at menarche, age at first full term pregnancy and parity, age at menopause, breastfeeding, ever use of contraceptive pills, ever use of menopausal hormonal therapy, physical activity index, alcohol consumption, smoking status, educational level, height, and energy intake

^1^Metabolically healthy/normal weight (BMI < 25 kg/m^2^ or Waist circumference <80 cm or Waist-to-hip ratio <0.8) plus within tertile 1 of C-peptide.

^2^Metabolically healthy/overweight or obese (BMI ≥ 25 kg/m^2^ or Waist circumference ≥80 cm or Waist-to-hip ratio ≥0.8) plus within tertile 1 of C-peptide.

^3^Metabolically unhealthy/normal weight (BMI < 25 kg/m^2^ or Waist circumference <80cm or Waist-to-hip ratio <0.8) plus above tertile 1 of C-peptide.

^4^Metabolically unhealthy/overweight or obese (BMI ≥ 25 kg/m^2^ or Waist circumference ≥80cm or Waist-to-hip ratio ≥0.8) plus above tertile 1 of C-peptide

| **Table S4: Association between metabolic health–defined body size phenotypes using anthropometric cut-points and breast cancer risk in postmenopausal women:**  **Upper tertile of C-peptide classified as metabolically unhealthy or C-peptide above the median or above the first quartile classified as metabolically unhealthy** | | | | |
| --- | --- | --- | --- | --- |
| Metabolic health–defined body size phenotypes | **Metabolically healthy** | | **Metabolically unhealthy** | |
|  | **Normal weight^1^** | **Overweight/Obesity^2^** | **Normal weight^1^** | **Overweight/Obesity^2^** |
| ***Upper tertile classified as metabolically unhealthy*** |  |  |  |  |
| **Metabolic Health/ BMI Definition** |  |  |  |  |
| N cases/controls | 152/330 | 223/415 | 57/137 | 178/248 |
| Model 1 OR (95% CI) | 1 [reference] | 1.18 (0.91-1.53) | 0.94 (0.63-1.38) | **1.62 (1.20-2.19)** |
| Model 2 OR (95% CI) | 1 [reference] | 1.24 (0.95-1.62) | 0.90 (0.60-1.34) | **1.75 (1.27-2.41)** |
| **Metabolic Health/ WC Definition** |  |  |  |  |
| N cases/controls | 158/337 | 217/408 | 58/113 | 177/272 |
| Model 1 OR (95% CI) | 1 [reference] | 1.19 (0.91-1.54) | 1.16 (0.78-1.72) | **1.47 (1.09-1.99)** |
| Model 2 OR (95% CI) | 1 [reference] | 1.17 (0.89-1.54) | 1.12 (0.74-1.69) | **1.50 (1.10-2.04)** |
| **Metabolic Health/ WHR Definition** |  |  |  |  |
| N cases/controls | 172/348 | 203/397 | 78/137 | 157/248 |
| Model 1 OR (95% CI) | 1 [reference] | 1.04 (0.80-1.35) | 1.21 (0.83-1.76) | 1.32 (0.98-1.77) |
| Model 2 OR (95% CI) | 1 [reference] | 1.04 (0.79-1.37) | 1.19 (0.81-1.75) | **1.35 (1.00-1.84)** |
| ***C-peptide>median classified as metabolically unhealthy*** |  |  |  |  |
| **Metabolic Health/ BMI Definition** |  |  |  |  |
| N cases/controls | 125/265 | 168/298 | 84/202 | 233/365 |
| Model 1 OR (95% CI) | 1 [reference] | 1.23 (0.92-1.65) | 0.89 (0.62-1.27) | **1.35 (1.01-1.81)** |
| Model 2 OR (95% CI) | 1 [reference] | 1.25 (0.92-1.70) | 0.82 (0.57-1.18) | **1.41 (1.04-1.91)** |
| **Metabolic Health/ WC Definition** |  |  |  |  |
| N cases/controls | 129/276 | 164/287 | 87/174 | 230/393 |
| Model 1 OR (95% CI) | 1 [reference] | 1.29 (0.96-1.73) | 1.09 (0.76-1.55) | 1.29 (0.97-1.73) |
| Model 2 OR (95% CI) | 1 [reference] | 1.25 (0.93-1.70) | 1.02 (0.70-1.47) | 1.27 (0.94-1.73) |
| **Metabolic Health/ WHR Definition** |  |  |  |  |
| N cases/controls | 144/278 | 149/285 | 106/207 | 211/360 |
| Model 1 OR (95% CI) | 1 [reference] | 1.02 (0.75-1.37) | 0.99 (0.71-1.39) | 1.13 (0.85-1.51) |
| Model 2 OR (95% CI) | 1 [reference] | 1.00 (0.73-1.35) | 0.93 (0.66-1.31) | 1.12 (0.84-1.51) |
| ***C-peptide>first quartile classified as metabolically unhealthy*** |  |  |  |  |
| **Metabolic Health/ BMI Definition** |  |  |  |  |
| N cases/controls | 35/73 | 26/44 | 174/394 | 375/619 |
| Model 1 OR (95% CI) | 1 [reference] | 1.26 (0.68-2.34) | 0.90 (0.57-1.40) | 1.25 (0.81-1.91) |
| Model 2 OR (95% CI) | 1 [reference] | 1.40 (0.74-2.66) | 0.94 (0.59-1.48) | 1.39 (0.89-2.16) |
| **Metabolic Health/ WC Definition** |  |  |  |  |
| N cases/controls | 34/82 | 27/35 | 182/368 | 367/645 |
| Model 1 OR (95% CI) | 1 [reference] | **1.98 (1.04-3.74)** | 1.16 (0.75-1.81) | 1.39 (0.91-2.13) |
| Model 2 OR (95% CI) | 1 [reference] | **2.12 (1.09-4.13)** | 1.25 (0.79-1.97) | 1.48 (0.95-2.31) |
| **Metabolic Health/ WHR Definition** |  |  |  |  |
| N cases/controls | 43/80 | 18/37 | 207/405 | 342/608 |
| Model 1 OR (95% CI) | 1 [reference] | 0.96 (0.49-1.86) | 0.94 (0.62-1.43) | 1.04 (0.70-1.56) |
| Model 2 OR (95% CI) | 1 [reference] | 1.04 (0.52-2.06) | 1.00 (0.65-1.54) | 1.11 (0.73-1.68) |

Model 1 was adjusted on matching criteria only and included age at blood collection, time of day at blood collection and fasting status at blood collection.

Model 2 was adjusted on matching factors, with additional adjustment for age at menarche, age at first full term pregnancy and parity, age at menopause, breastfeeding, ever use of contraceptive pills, ever use of menopausal hormonal therapy, physical activity index, alcohol consumption, smoking status, educational level, height, and energy intake.

^1^Metabolically healthy/normal weight (BMI < 25 kg/m^2^ or Waist circumference <80 cm or Waist-to-hip ratio <0.8) plus within tertile 1 of C-peptide.

^2^Metabolically healthy/overweight or obese (BMI ≥ 25 kg/m^2^ or Waist circumference ≥80 cm or Waist-to-hip ratio ≥0.8) plus within tertile 1 of C-peptide.

^3^Metabolically unhealthy/normal weight (BMI < 25 kg/m^2^ or Waist circumference <80cm or Waist-to-hip ratio <0.8) plus above tertile 1 of C-peptide.

^4^Metabolically unhealthy/overweight or obese (BMI ≥ 25 kg/m^2^ or Waist circumference ≥80cm or Waist-to-hip ratio ≥0.8) plus above tertile 1 of C-peptide

| **Table S5: Association between metabolic health–defined body size phenotypes using different anthropometric cut-points (88 cm for WC and 0.88 for WHR) and breast cancer risk in postmenopausal women** | | | | |
| --- | --- | --- | --- | --- |
| Metabolic health–defined body size phenotypes | **Metabolically healthy** | | **Metabolically unhealthy** | |
|  | **Normal weight^1^** | **Overweight/Obesity^2^** | **Normal weight^1^** | **Overweight/Obesity^2^** |
| **Metabolic Health/ WC Definition** |  |  |  |  |
| N cases/controls | 140/302 | 33/70 | 251/484 | 186/274 |
| Model 1 OR (95% CI) | 1 [reference] | 1.08 (0.68-1.74) | 1.16 (0.88-1.52) | **1.53 (1.13-2.06)** |
| Model 2 OR (95% CI) | 1 [reference] | 1.06 (0.65-1.72) | 1.16 (0.88-1.54) | **1.55 (1.13-2.12)** |
| **Metabolic Health/ WHR Definition** |  |  |  |  |
| N cases/controls | 155/330 | 18/42 | 369/638 | 68/120 |
| Model 1 OR (95% CI) | 1 [reference] | 0.87 (0.49-1.57) | 1.27 (0.99-1.62) | 1.21 (0.83-1.74) |
| Model 2 OR (95% CI) | 1 [reference] | 0.94 (0.52-1.70) | **1.29 (1.00-1.67)** | 1.23 (0.84-1.80) |

Model 1 was adjusted on matching criteria only and included age at blood collection, time of day at blood collection and fasting status at blood collection.

Model 2 was adjusted on matching factors, with additional adjustment for age at menarche, age at first full term pregnancy and parity, age at menopause, breastfeeding, ever use of contraceptive pills, ever use of menopausal hormonal therapy, physical activity index, alcohol consumption, smoking status, educational level, height, and energy intake.

^1^Metabolically healthy/normal weight (Waist circumference < 88 cm or Waist-to-hip ratio < 0.88) plus within tertile 1 of C-peptide.

^2^Metabolically healthy/overweight or obese (Waist circumference ≥ 88 cm or Waist-to-hip ratio ≥ 0.88) plus within tertile 1 of C-peptide.

^3^Metabolically unhealthy/normal weight (Waist circumference < 88 cm or Waist-to-hip ratio < 0.88) plus above tertile 1 of C-peptide.

^4^Metabolically unhealthy/overweight or obese (Waist circumference ≥ 88 cm or Waist-to-hip ratio ≥ 0.88) plus above tertile 1 of C-peptide.
